# Interaction of Br with Cu(100) surface: adsorption and thermodynamics stability study

**DOI:** 10.1039/d5ra02406d

**Published:** 2025-08-11

**Authors:** Ali Marashdeh, Sherin A. Saraireh, Niveen W. Assaf, Suzan K. Alghamdi, Akef T. Afaneh, Wissam Helal, Mohammednoor Altarawneh

**Affiliations:** a Department of Chemistry, Faculty of Science, Al-Balqa Applied University Al-Salt 19117 Jordan a.marashdeh@bau.edu.jo; b Physics Department, Faculty of Sciences, Taibah University Al-Madeina Al-Munawarah Saudi Arabia SSARAIREH@taibahu.edu.sa; c Physics Department, Faculty of Sciences, Al-Hussein Bin Talal University Ma'an Jordan; d Chemical Engineering Department, Faculty of Engineering Technology, Al-Balqa Applied University Al-Salt 19117 Jordan; e Department of Chemistry, The University of Jordan Amman 11 942 Jordan; f Department of Chemical and Petroleum Engineering, United Arab Emirates University Sheikh Khalifa Bin Zayed Street Al-Ain 15551 United Arab Emirates

## Abstract

The interaction between halogen species and metal surfaces plays a crucial role in numerous surface-driven processes. The adsorption behavior and thermodynamic stability of bromine (Br) atoms on the Cu(100) surface using density functional theory (DFT) with van der Waals (vdW) corrections and *ab initio* atomistic thermodynamics is investigated. Surface adsorption was analyzed across three high-symmetry sites-top, bridge, and hollow-at various coverages from (0.11 to 1.00) ML. van der Waals forces significantly influence binding energy, particularly at low coverage. Hollow sites are identified as the most energetically favorable. The hollow site emerged as the most energetically favorable across most coverages, with the 0.75 ML configuration being particularly stable. Electronic density of states (DOS) analysis revealed strong Br–Cu hybridization and significant alterations in the Cu(3d) and Br(4p) states, especially at higher coverages. Thermodynamic phase diagrams demonstrated that increasing Br chemical potential leads to progressively more stable Br/Cu(100) surface configurations, shifting from clean Cu to partial and then full monolayer adsorption.

## Abstract 2

Inclusion of van der Waals corrections significantly enhances the binding energies of Br on the Cu(100) surface, with higher Br chemical potential promoting more stable Br/Cu(100) configurations, among which the hollow site at 0.75 ML coverage is the most favorable.

## Graphical abstract

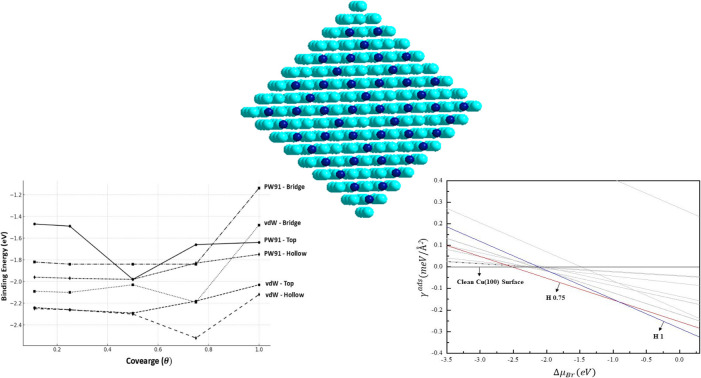

## 1. Introduction

The chemistry of halogens on metal surfaces has sparked considerable research interest.^1–11^ It has numerous industrial and technological applications, including corrosion and catalysis.^12–16^ They function as redox shuttles and catalytic modifiers.^17^ Since the 1970s, various studies have been conducted to gain a thorough understanding of halogen–metal interactions at the atomic scale. Halogens, including bromine, iodine and fluorine, exhibit robust interactions with metallic surfaces.^18^ Often leads to the formation of metal halides through chemisorption or direct chemical reactions. Such reactions can significantly influence surface morphology, electrical structure, and chemical reactivity.^13^ Consequently, it holds significant scientific and technological importance. Copper is a crucial industrial material owing to its minimal resistivity and resistance to electromigration.^19,20^ The interaction of halogen and copper is particularly significant due to its applications in catalysis and semiconducting industry, especially for low-temperature dry etching processes. Several studies have examined the interactions between bromine (Br_2_), chlorine (Cl_2_) and copper (Cu).^21–34^ The process of copper etching utilizing chlorine and bromine involves two stages: initial halogenation to generate bulk copper halide, followed by sublimation.^28,35–38^ The formation of CuCl and their adsorption rates are constrained, with the reaction rate being directly proportional to the reaction temperature.^35,39^

Winters^34^ investigated the exposure of the copper surface to chlorine and noted the development of the chlorine-chemisorped layers on Cu(100), whereas Park *et al.*^30^ and Richardson *et al.*^32^ reported that the Br_2_–Cu system functions similarly to Cl_2_ on Cu(100). Richardson *et al.*,^32^ state that following the detachment of Br_2_, a chemisorbed layer of Br atoms forms on the Cu surface, with saturation of the Br layers occurring after 2.5 L dosing.

Nakakura *et al.*^28^ employed TPD and LEED to investigate the interaction of Br_2_ with Cu(100). The overall process for copper manufacture commences with the synthesis of copper(i) bromide. CuBr was removed *via* sublimation, with desorption peaks seen at 870 K and 1000 K. At 325 K, the *c* (2 × 2) pattern was promptly seen upon the exposer of the Cu(100) surface to Br_2_. The Br–Br distance measured 3.62 Å in the CuBr layer, considerably less than the 4.02 Å distance seen in bulk CuBr.^40^ The configuration of the CuBr layer fluctuates with temperature; at 365 K, a *c* (2 × 2) pattern is seen, and the strength of the LEED patterns decreases as the sample is further heated beyond 365 K. The results further demonstrate that halide desorption is affected by the layer's thickness. Likewise, the reaction rate decreased as the temperature rose during the interaction of Br_2_ with Cu(100).

Nakakura *et al.*^28^ subsequently examined the interactions of Cl_2_ and Br_2_ with Cu(100) utilizing low-energy electron diffraction (LEED) and temperature-programmed desorption (TPD). It was determined that Br_2_ is superior for Cu etching relative to Cl_2_ as Br_2_ exhibits greater reactivity in halide formation than Cl_2_. From an industrial perspective, comprehending the individual reaction steps is essential; therefore, it is imperative to examine and characterize the atomic adsorption of bromine and chlorine on the copper surface *via* theoretical calculations.

To date, a number of studies have examined atomic and molecular chemisorption on copper surfaces.^27,40–47^*Ab initio* atomistic thermodynamics have been conducted for chlorine chemisorption on the Cu(001) surface at different chlorine pressures and temperatures to clarify the production of copper chloride CuCl.^43^ Based on the computed surface free energy, the 0.5 ML *c*(2 × 2)–Cl hollow sites were identified as the most stable configuration. At low pressures and elevated temperatures, the (1 and 2) ML adsorption arrangements facilitate the emergence of a bulk chlorine surface phase.

Along the same lines, Kenny *et al.*^44^ performed calculations utilizing density functional theory to examine the adsorption of Br on Cu(100) surface at low coverage. The four-fold hollow site represents the most stable location for Br atoms. The energy barrier for Br atoms transitioning between neighboring hollows through two-fold saddle points is around 0.23 eV, while the barrier for movement along 〈010〉 directions *via* the on-top site is 0.37 eV. The last result suggests that scanning tunneling microscopy (STM) images cannot be acquired at low coverage under room temperature conditions. Moreover, the authors associated the Br atoms in the hollow sites with Br atoms attached to the vacancy sites on the copper surface.^44^

This study utilized density functional theory (DFT) and *ab initio* atomistic thermodynamics to investigate the atomic adsorption of bromine on a Cu(100) surface. This was conducted to ascertain the thermodynamic stability of bromine adsorption under varying Br exposure (pressure and temperature) conditions at distinct sites on the Cu(100) surface, top (T), hollow (H), and bridge (B) sites, with bromine coverages of (0.11, 0.25, 0.5, 0.75, and 1) (ML). This study's DFT calculations use van der Waals correction function (vdE-DF),^48–50^ addressing long-term interactions.^50^ This work aimed to elucidate the synthesis process influenced by variable concentrations of bromine reducing agents at varied pressures and temperatures, and to examine the formation of CuBr.

## 2. Computational details

All computations were carried out using the *P*,^51–53^ which relies on periodic plane wave pseudopotential density functional theory (DFT). The exchange and correlation contributions of the atoms were outlined using the generalized gradient approximation (GGA) developed by Perdew *et al.* (PW91).^54^ To solve Kohn–Sham equations, the projected-augmented plane wave (PAW) method^55,56^ is implied to describe the electron-core interaction. Long-range interactions are accounted for by van der Waals (vdW) corrections (vdW-DFT)^48,49,57^ exchange–correlation functionals. The inclusion of the vdW-DFT is vital for enhancing the binding energies of both physisorbed and chemisorbed species, as mentioned in earlier studies.^57–59^ Dipole corrections are applied for the asymmetric slab computations, using the upper surfaces of the slabs.

The Cu(100) surface is modeled by a 2 × 2 supercell comprising 6 layers (27 Cu atoms) and a 3 × 3 supercell that has 6 layers (48 Cu atoms). A test on the structure (H-0.25) by a 2 × 2 supercell comprising 8 layers (36 Cu atoms) results in a total energy change of merely 0.9 meV and a binding energy change of 1.3 meV. A vacuum layer of 20 Å is employed to reduce the interaction between adjacent slabs. To identify the most favorable sites, the four uppermost layers along with the adsorbate atoms were permitted to relax, while the remaining surface was kept in their bulk positions.

Geometric optimizations for all surfaces are performed using a Monkhorst–Pack *κ*-points sampling for the Brillouin-zone integration (4 × 4 × 1),^60^ after the *κ*-point mesh has been varied from (4 × 4 × 1) to (8 × 8 × 1). Based on a series of energy cutoff convergence tests, the energy cutoff for the plane wave basis was set to be 400 eV.^61^

Relaxations (*d*_ab_%) between layers a and b are determined using the bulk spacing, *d*_i_, which is associated with the surface spacing dab:1
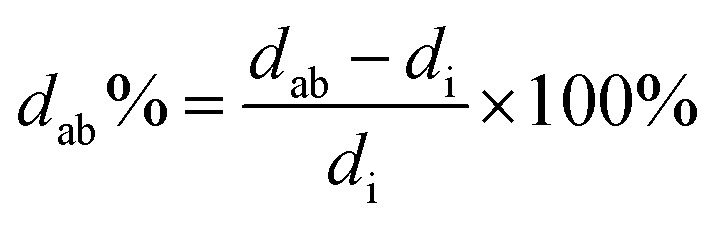


The binding energies, *E*_b_, of the adsorbed Br atoms have been calculated using the equations:2
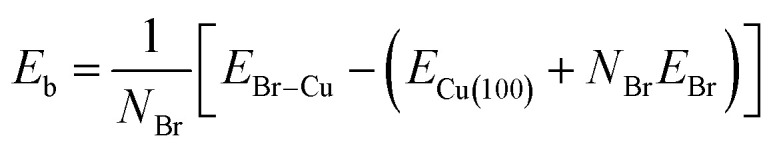
where *N*_Br_ is the number of adsorbed bromine atoms and *E*_Cu(100)_, *E*_Br_ and *E*_Br–Cu_ signify the total energies of a clean surface, an isolated bromine (in its natural state), and the Br/Cu(100) adsorption system, respectively. Positive binding energies, *E*_b_, indicates that the adsorption is endothermic, and negative value indicates the exothermic characteristic of the adsorption.

*Ab initio* atomistic thermodynamics calculations^62–66^ have been carried out to assess the stability of the Cu(100) surface following exposure to a bromine gaseous environment. These calculations were achieved to generate the temperature–pressure thermodynamic stability phase diagram for all possible configurations at varying coverages. The bromine chemical potential (Δ*μ*_Br_), as a function of the gas temperature (*T*) and partial pressure (*P*) is utilized to carry out these calculations, after which the surface free energy is computed. At equilibrium, the minimum surface free energy (*γ*_ads_) indicates the most thermodynamically favored structure.

The *γ*_ads_ is given by:3
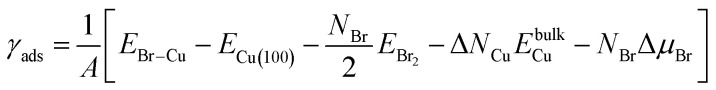
where *A* is the cell surface-area, Δ*N*_Cu_ is the difference between the number of Cu atoms of the Br/Cu system and the clean surface Cu(100).

And

4 Δ*μ*_Br_ = *μ*_Br_ − ½*E*_Br_2__

where *μ*_Br_ represents bromine chemical potential. The Δ*μ*_Br_ dependent on *T* and *P* by:5
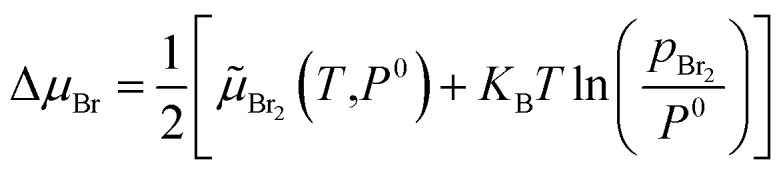
where *P*^0^ is the atmospheric pressure, and *μ̃*_Br_2__(*T*, *P*^0^) accounts for the contribution from the rotations and vibrations of a Br_2_ molecule in addition to the ideal-gas entropy at 1 atm. The latter can be found in thermodynamics tables.^66^

## 3. Results and discussion

### 3.1. Bulk Cu and clean Cu(100) surface

**Fig. 1:**
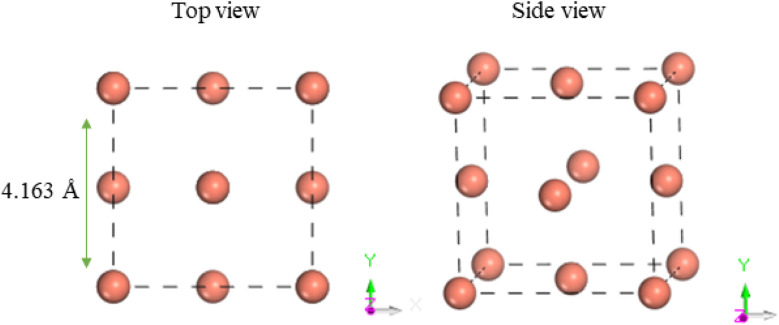
Top and side views of the unit cell of bulk Cu.

Fig. 1 presents the optimized unit cell of copper, which adopts a face-centered cubic (FCC) crystalline structure. The calculated lattice constant of 3.61 Å closely aligns with the experimental value of 3.615 Å. The (100) surface of Cu exhibits significant chemical reactivity with its environment. The calculated bulk separation along the 100 direction is 1.83 Å, aligning well with the theoretical results of 1.82 Å.^46^

**Fig. 2:**
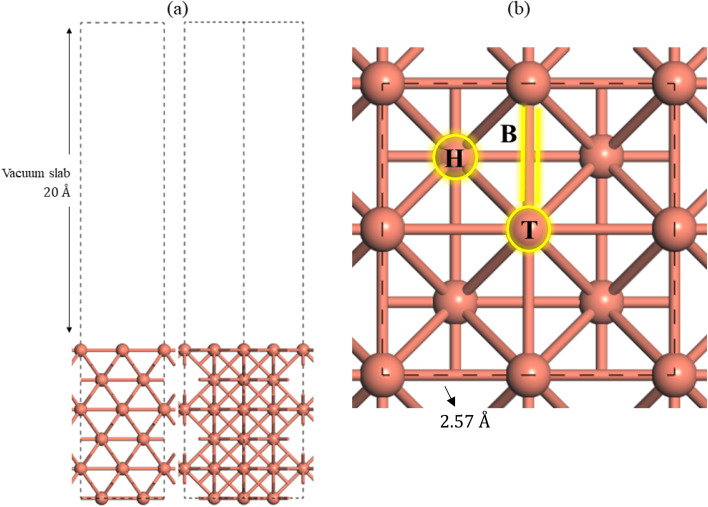
(a) Vacuum slab of the Cu(100) (2 × 2). (b) Top view of the adsorption sites on the Cu(100) surface.

Fig. 2 depicts the optimized (100) surface, exhibiting Cu–Cu bond lengths of 2.57 Å, consistent with the findings of Wang *et al.* at 2.57 Å.^67^Table 1 presents several computed properties for bulk copper and Cu(100) surface. The equilibrium lattice constant for the bulk copper was determined by minimizing energy with respect to the volume of the unit cell. The equilibrium lattice constants for the bulk Cu are *a*_Cu_ = 3.685 Å, whereas the experimental and the calculated measurements are 3.61 Å (ref. 68) and 3.64 Å,^46^ respectively. Indicating a strong correlation between our calculated value and the experimental value reported and calculated value with the discrepancy being under 2% and 1.2%, respectively.

**Table 1:** Measured and literature properties for the bulk Cu and the Cu(100) surface, *a*_Cu_: the equilibrium lattice constants for the bulk Cu, *d*_12_: relaxation of the first and second layer for the bulk Cu, and *d*_23_: relaxation of the second and third layers concerning the bulk Cu

	Current study	Previous DFT-GGA studies	Previous experimental data
*a*_Cu_	3.685 Å	3.64 Å (ref. 46)	3.61 Å (ref. 68)
*d*_12_	−1.56%	−2.3%,^46^ −2.6% (ref. 69)	−2.1% (ref. 70 and 71)
*d*_23_	+1.02%	+1%,^46^ +0.9% (ref. 69)	+0.5% (ref. 70 and 71)

The relaxation value calculated for the first and second layers relative to the bulk Cu (*d*_12_) of (−1.56%) shows a minor reduction compared to the bulk. This value is slightly lower than previous DFT-GGA computations^46,69^ and marginally higher than the experimental results.^70,71^ Moreover, our calculated relaxation of the second and third layers for bulk Cu (*d*_23_) at (+1.02%) exceeds the experimental measurement of (+0.5%)^70,71^ and in consistent with previous earlier DFT-GGA calculations of (+1%)^46^ and (+0.9%).^69^

The Cu(100) surface features three potential sites for gas adsorption, identified as T (the top site on the upper Cu atom), H (the hollow site between four Cu atoms), and B (the bridge site situated between two Cu atoms) as depicted in Fig. 2b. Six layers of the Cu(100) surface was utilized due to the disparity between odd and even layers; thus, we employed six layers with each pair of layers repeated three times.

### 3.2. On-surface adsorptions of Br on Cu(100) surface

**Fig. 3:**
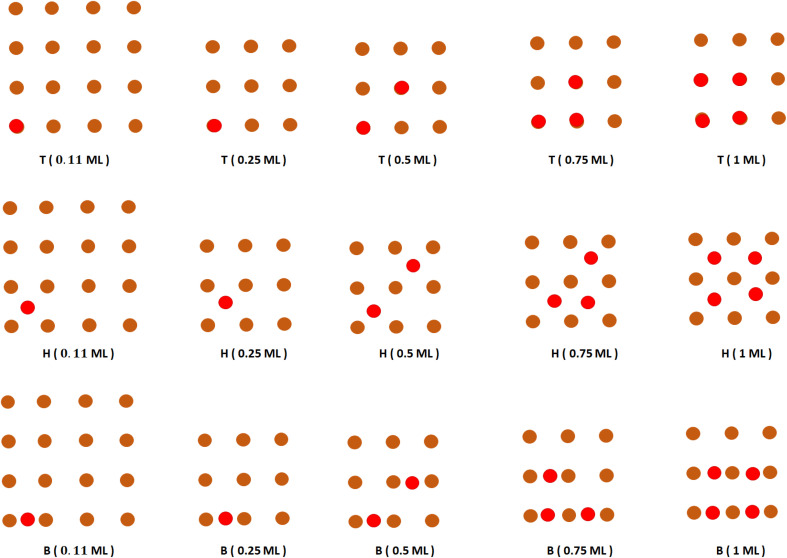
Top views of on-surface Br atoms at different coverages on top, hollow and bridge sites. Brown atoms represent the first layer of the Cu(001) surface, red atoms represent the bromine atoms.

For the on-surface adsorptions of Br on Cu(100) surface, three potential adsorption sites (T, B, and H) are identified based on Fig. 2b and were considered for atomic bromine adsorption at coverages of (
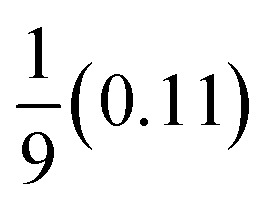
, ¼(0.25), ½(0.5), ¾(0.75), and 1) ML (as shown in Fig. 3). The binding (*E*_b_) and chemisorption (*E*_chem._) energies are given in Table 2 for the different sites of Br/Cu(100) configurations (the values are negative and the lowest value corresponds to the most stable configurations).

**Table 2:** The binding energy (*E*_b_) of the Br on Cu(100) surface, are calculated using eqn (1) for different coverages (*θ*). The enhancement factor is 
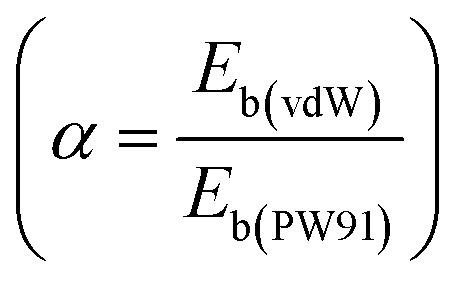

System (site)	*θ* (ML)	Ref. 44			Current					
		Cell size	Number of layers	PW91	Cell size	Number of layers	*E*_b_ (eV)		Δ*E* = *E*(PW91) − *E*(vdW)	*α*
							PW91	vdW		
T	0.11	3 × 3	7	−1.49	3 × 3	6	−1.47	−2.24	0.77	1.524
T	0.25	2 × 2	7	−1.57	2 × 2	6	−1.49	−2.26	0.77	1.517
T	0.50	—	—	—	2 × 2	6	−1.98	−2.29	0.31	1.157
T	0.75	—	—	—	2 × 2	6	−1.66	−2.18	0.52	1.313
T	1.00	—	—	—	2 × 2	6	−1.64	−2.03	0.39	1.238
B	0.11	3 × 3	7	−1.83	3 × 3	6	−1.82	−2.09	0.27	1.148
B	0.25	2 × 2	7	−1.71	2 × 2	6	−1.84	−2.10	0.26	1.141
B	0.50	—	—	—	2 × 2	6	−1.84	−2.03	0.19	1.103
B	0.75	—	—	—	2 × 2	6	−1.84	−2.19	0.35	1.190
B	1.00	—	—	—	2 × 2	6	−1.14	−1.48	0.29	1.298
H	0.11	3 × 3	7	−1.98	3 × 3	6	−1.96	−2.25	0.29	1.148
H	0.25	2 × 2	7	−1.94	2 × 2	6	−1.97	−2.26	0.29	1.147
H	0.50	—	—	—	2 × 2	6	−1.98	−2.30	0.32	1.162
H	0.75	—	—	—	2 × 2	6	−1.83	−2.52	0.69	1.377
H	1.00	—	—	—	2 × 2	6	−1.75	−2.12	0.37	1.211

The PW91 exchange–correlation functional has been used at low coverages to improve comparisons with ref. 44. This study produces binding energies that are slightly lower, possibly because of variations in vertical relaxation or dipole correction adjustments. The discrepancies between the study cited in ref. 44 and the present investigation span a range of −0.13 to +0.08 eV, a figure that falls within an acceptable margin of error for density functional theory calculations. The recent study's slight decrease in slab layers results in slightly weaker binding in the majority of cases, particularly at top and hollow sites. Current data indicates a more complex relaxation phenomenon at bridge sites, or alternatively, varying convergence standards impact these geometries.

The agreement between the two datasets indicates that the PW91 functional is a reliable choice for simulating, Br/Cu(100) with differences in adsorption energies suggesting a substantial reliance on computational parameters, particularly slab thickness and relaxation depth. The van der Waals correction consistently enhances the magnitude of the binding energy, signifying a stronger adsorption. The enhancement factor (*α*) is consistently higher than 1, which provides further evidence that van der Waals contributions significantly enhance the binding. The enhancement is most pronounced at low coverage levels and for hollow sites, which derive greater benefit from the dispersion interactions because of increased surface contact.

The van der Waals correction exerted the largest effect of approximately 0.77 eV at the lowest coverage level. At higher coverage levels, the effect is reduced, mostly due to the lateral repulsion between the adsorbates. The vdW effect is weaker at the bridge location than at the upper location, but it still benefits from the corrections made to froth dispersion. The correction increases substantially at complete coverage (*θ* = 1 ML) as a result of crowding effects. Websites with empty space show a non-linear response to corrections for van der Waals forces, with a notable impact at 0.75 ML, likely resulting from optimal packing or collective polarization effects of the substrate. The enhanced influence of van der Waals forces at lower coverage is attributed to the relatively isolated Br atoms, as a result of which long-range interactions between Br and surface Cu atoms are not hindered by surrounding adsorbates.

The data in Table 2 show that vdW corrections increase the binding strength across all configurations, with the most significant improvements occurring at low coverage and on top sites. The limitations of the plain PW91 method become apparent when it comes to improve adsorption in systems where dispersion forces are significant, thus supporting the application of vdW-inclusive methods in surface science simulations.

**Fig. 4:**
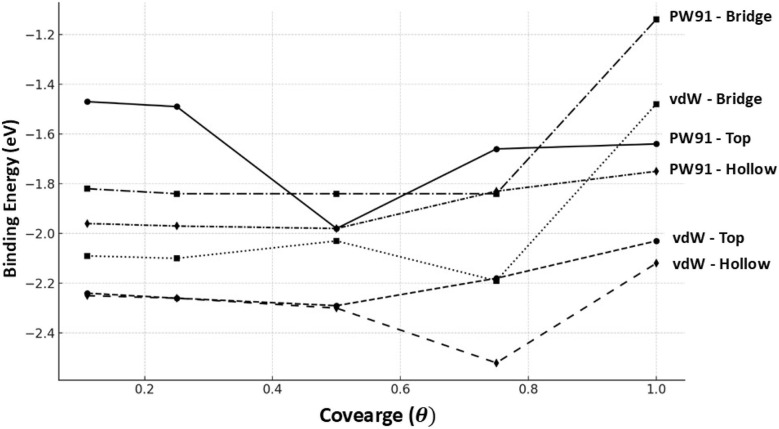
Comparison the PW91 and vdW binding energies across different coverages for the top, bridge, and hollow sites.

The relationship between binding energy (*E*_b_) and surface coverage is shown clearly in Fig. 4, as calculated with the PW91 functional. With increasing surface coverage, the binding energy becomes progressively more negative, indicating a significantly stronger adsorption of Br atoms on the Cu(100) surface. A consistent pattern is observed across the three types of on-surface adsorption sites – top (T), bridge (B), and hollow (H) – with a direct correlation between binding energy and coverage. Even at the lowest coverage of 0.11 ML, a small departure from the predicted pattern becomes apparent, particularly at the top site, where binding is less strong than at higher coverages. Consistently, the empty site exhibits the lowest binding energy at various coverages, further solidifying it as the preferred site in terms of energy within the PW91 framework. The binding energies of the bridge site are nearly identical to those of the hollow site at (0.11, 0.50, 0.75, and 1) ML. In contrast to the top site, it shows a weaker adsorption across all coverage values, which highlights the site dependence of the adsorption strength.

The incorporation of van der Waals interactions, as shown in Fig. 4, reveals a direct correlation between binding energy (*E*_b_) and coverage, even when using the vdW-corrected functional. This interaction notably increases the magnitude of (*E*_b_) for all adsorption sites, namely top (T), bridge (B), and hollow (H), ultimately resulting in a substantial enhancement of adsorption strength in comparison to the PW91 results. The effect is most noticeable at low coverage levels (0.11 ML), particularly at the top site, where the dispersion contribution has a significant influence (Fig. 4).

The hollow site configuration is the most energetically advantageous across various coverages, as indicated by the PW91 trends, with the bridge site showing comparable binding energies to the hollow site at (0.11, 0.50, 0.75, and 1.00) ML. Although it remains weaker in terms of binding energy compared to the others, the top site experiences the most significant improvement from vdW corrections, thereby reducing the difference in energy between sites. The vdW-corrected data highlights the significance of dispersion forces in enhancing modelling the adsorption behavior of Br on Cu(100), particularly at low surface concentrations and for low-coordination sites.

Table 3 provides concise explanations of all optimum structures for the H, B, and T configurations at various coverages. At the H site, it was observed that at (0.25 ML), (0.5 ML), (0.75 ML) and (1 ML), 100%, 100%, 67% and 75% of Br atoms, respectively, migrates to the B sites. At the T site and across all coverages of 0.11, 0.5, 0.75 and 1 ML, 100% of the Br atoms transitioned to the B site, whereas only the Br atoms at 0.25 ML remained stable at the T site. It is observed that nearly all Br atoms are stable at the B location.

**Table 3:** Characterizations for the optimized structures at various coverage (*θ*)

Site	*θ* (ML)	Br atom	First layer's Cu atoms
T	0.11	100% Br atom moved to B site	All Cu atoms remain in the first layer
	0.25	Stable at T site	One Cu atoms of the first layer moved to the second layer
	0.5	100% Br atom moved to B site	All Cu atoms remain in the first layer
	0.75	100% Br atom moved to B site	All Cu atoms remain in the first layer
	1	100% Br atom moved to B site	All Cu atoms remain in the first layer
B	0.11	Stable at B site	All Cu atoms remain to reside in the first layer
	0.25	Stable at B site	All Cu atoms stable in the first layer
	0.5	Stable at B site	One Cu atom of the first layer moved to the second layer
	0.75	Stable at B site. Two Br layers are produced	One Cu atom of the first layer moved to the second layer
	1	Stable at B site. Two Br layers are produced	One Cu atom of the first layer moved to the second layer
H	0.11	Stable at H site	All Cu atoms remain stable in the first layer
	0.25	100% Br atom moved to B site	All Cu atoms are stable in the same height of the Cu's first layer height (not moved to the second layer)
	0.5	100% Br atom moved to B site	One Cu atom of the first layer moved to the second layer
	0.75	33% Br atom stayed at H site. 67% Br atom moved to B site. Two Br layers are produced	One Cu atom of the first layer moved up to Br-layer
	1	25% Br atom stayed at H site. 75% Br atom moved to B site. Two Br layers are produced	All Cu atoms remain in the first layer

Modifications are noted in the first layer of the Cu atoms; for coverages, (0.5–1) ML of the B site, one of the Cu atom resides in the second layer, a phenomenon exclusively observed at the H-0.5 ML and the T-0.25 ML. The optimized parameters of the preferred configuration H (0.25 ML) reveal that the Br atom migrates from the H site to a position nearer to the T site, while the Cu atom relocates to the B site. This behavior implies that the Br atom is situated in the position of one of the Cu atoms in the first layer, which is consistent with the findings of Kenny *et al.*^44^

**Fig. 5:**
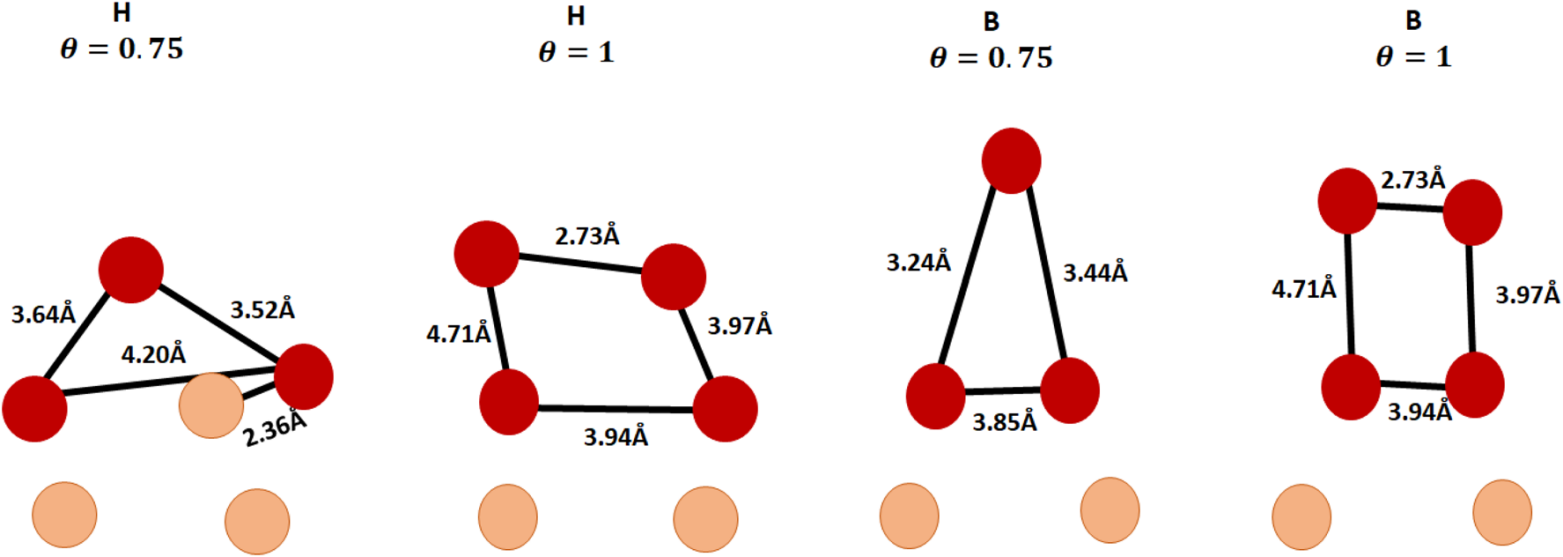
The optimized structures H (0.75–1) ML and B (0.75–1) ML, the atoms are shown in the diagram as brown spheres signifying the atoms of the Cu surface, while the red spheres represent bromine atoms.

In configuration H (0.75 ML) (Fig. 5); the Br atoms disrupt the Cu–Cu bonds between the surface and subsurface layers, resulting in the formation of two Br thin film layers atop the slab; the first layer comprises CuBr and an extra Br atom, whereas the second layer consists of only one Br atom. The distance between the film and the slab is 1.99 Å. The Cu–Br bond length in the film's first layer measures 2.36 Å, consistent with the estimated value for bulk CuBr (2.47 Å). The Br–Br distance measured at 4.20 Å in the first layer is greater than the 4.02 Å distance observed in bulk CuBr,^40^ but the distance of (3.52–3.64) Å between Br atoms in different layers is shorter than that the 4.02 Å distance in bulk CuBr.^40^ In the H (1 ML) configuration (Fig. 5); two layers of a thin film of Br are formed on the top of the slab.

The distance between the first layer of the film and the slab is 1.98 Å, while the distance between the two Br's layers is 3.18 Å. The Br–Br distance is measured at 3.94 Å between atoms in the first layer and 2.73 Å in the second layer, both of which are shorter than the 4.02 Å distance observed in bulk CuBr. In configuration B (0.75 ML) (Fig. 5), two bromines layers of a thin film develop on the top of the slab. The distance between the first layer of the film and the slab is 1.93 Å, while the distance between the two Br layers is 2.18 Å. The Br–Br distance was measured at 3.85 Å in the first layer and (3.24–3.44) Å between two Br atoms in different layers, both of which are shorter than the 4.02 Å distance in bulk CuBr.

In configuration B (1 ML) (Fig. 5), two thin film layers of bromine are formed on the top of the slab. The distance between the first layer of the film and the slab is 1.98 Å, whereas the separation between the two Br layers is 3.17 Å. The measured Br–Br distance was found to be (2.73–3.94) Å within the same layer and (3.94–4.71) Å between Br atoms in different layers, where one exceeding the 4.02 Å distance observed in bulk CuBr, while the other is shorter.

Table 3 indicates that H sites at (0.11, 0.25, and 0.5) ML are transferred to B sites for the corresponding coverages, and the final configurations for (H-0.75 ML) remains unchanged, with 67% of the atoms relocated to B sites. According to the latest findings, the most stable site for a Br on the Cu(100) surface is occurring at the B (0.25 ML), followed by B (0.5 ML).

Analysis of the binding energy of the Br/Cu(100) system, in comparison to analogous values obtained for the Cl/Cu(100) system,^42^ reveals that Br demonstrates greater reactivity relative to Cl in its interactions with Cu(100), which aligns with the experimental findings reported by Nakakura *et al.*^28^

Table 4 summarizes structural parameters for Br/Cu(100) across different coverages (*θ*, in ML). The key structural trends associated with coverage and site-specific behavior. *d*_12_ (relaxation), denotes the relaxation between first and second copper layers, with H and B sites exhibiting negative relaxation for (0.11, 0.25, 0.5, and 1) ML, suggesting contraction for the first two layers, except for (0.75 ML). The T site exhibits positive changes, implying enlarged for the first two layers. The B site show minimal changes for (0.11, 0.5, 0.75, and 1) ML.

**Table 4:** *d*_12_: is the average relaxation (as a percentage) between first and second layers, *d*(Br–Cu): the bond length between Br atom(s) and the nearest Cu atoms. *d*(Br–Br): the bond length between nearest Br atoms. *Y*: the average of the heights of Br atoms above the first layer. *H*: the height between the highest and lowest atoms in the first layer of the surface Cu atoms

System	*θ* (ML)	*d*_12_ (%)	*d*(Br–Cu) (Å)	*d*(Br–Br) (Å)	*Y*(Å)	*H*(Å)
T	0.11	+2.45	1.89	—	1.45	0.02
	0.25	+5.65	2.39	—	1.44	0.01
	0.50	+0.65	2.22	3.83	1.38	0.02
	0.75	+2.55	2.31	3.79	1.42	0.03
	1.00	+2.45	2.23	3.54	1.47	0.01
B	0.11	−0.07	2.42	—	2.07	0.11
	0.25	−3.75	2.56	—	1.84	0.10
	0.50	−0.06	2.58	3.87	1.61	0.03
	0.75	+0.22	2.48	3.24	1.51	0.05
	1.00	−0.35	2.09	2.73	1.60	0.01
H	0.11	−3.80	2.60	—	1.86	0.08
	0.25	−3.22	2.18	—	1.92	0.13
	0.50	−0.57	2.35	2.75	1.99	0.04
	0.75	+1.24	2.36	3.64	1.93	0.27
	1.00	−1.51	2.41	2.73	1.96	0.35

The bond length of *d*(Cu–Br) varies among sites and increases with coverage, particularly at B sites. *d*(Br–Br) (interatomic distance) the H and T sites are exhibiting consistency, whereas the B sites demonstrate more variability. The average Br height (*Y*) is highest for H sites at most coverages, particularly above 0.25 ML. The height range in the Cu layer (H) is minimal but increases substantially for all sites at 1 monolayer.

**Fig. 6:**
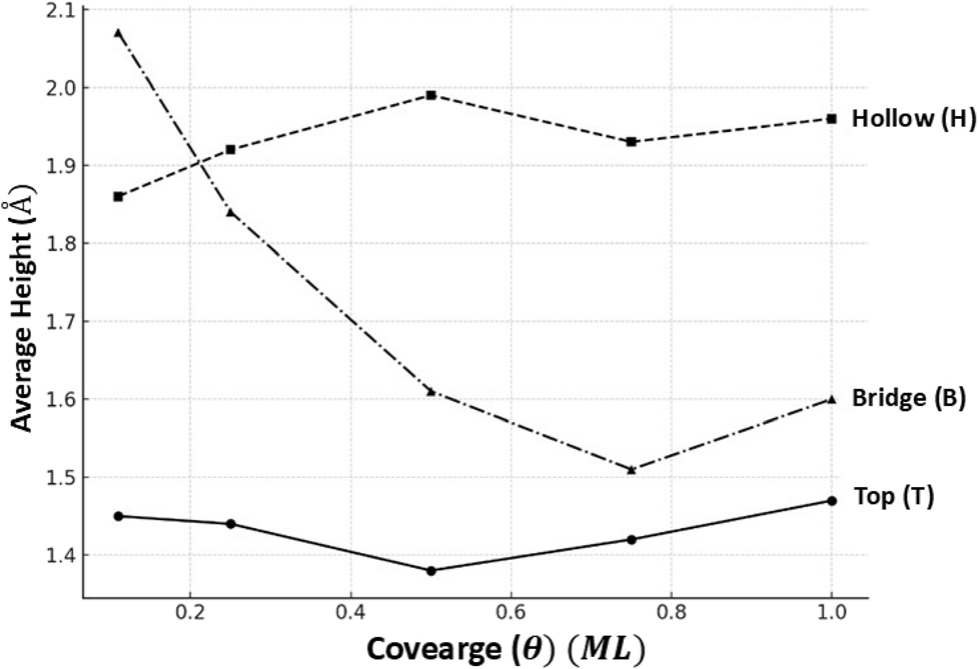
Variation of the average height (*Y*) of Br atoms above the first Cu layer as a function of surface coverage for different on-surface adsorption sites; top (T), hollow (H), and bridge (B).

Fig. 6 shows the change in the average vertical distance (*Y*) of Br atoms from the Cu(100) surface as a function of coverage for the three different adsorption sites: top (T), hollow (H), and bridge (B). At a low coverage of 0.11 ML, the bridge site labelled (B) displays the highest adsorption height, indicating a weaker interaction with the substrate at this point, whereas the top (T) and hollow (H) sites show a closer proximity to the surface. As the level of coverage increases (theta is greater than or equal to 0.25 ML), the average height at the hollow site consistently surpasses that at the top site, suggesting intensified repulsive interactions or spatial constraints at higher densities. Notably, the bridge site displays a downward trend in height as coverage increases, stabilizing at around 1.5–1.6 Å, which could indicate structural rearrangement or stronger bonding at denser surface populations. The relationship between the geometry of the adsorption site and the surface coverage is crucial in determining the vertical alignment of Br atoms.

### 3.3. Electronic structure

**Fig. 7:**
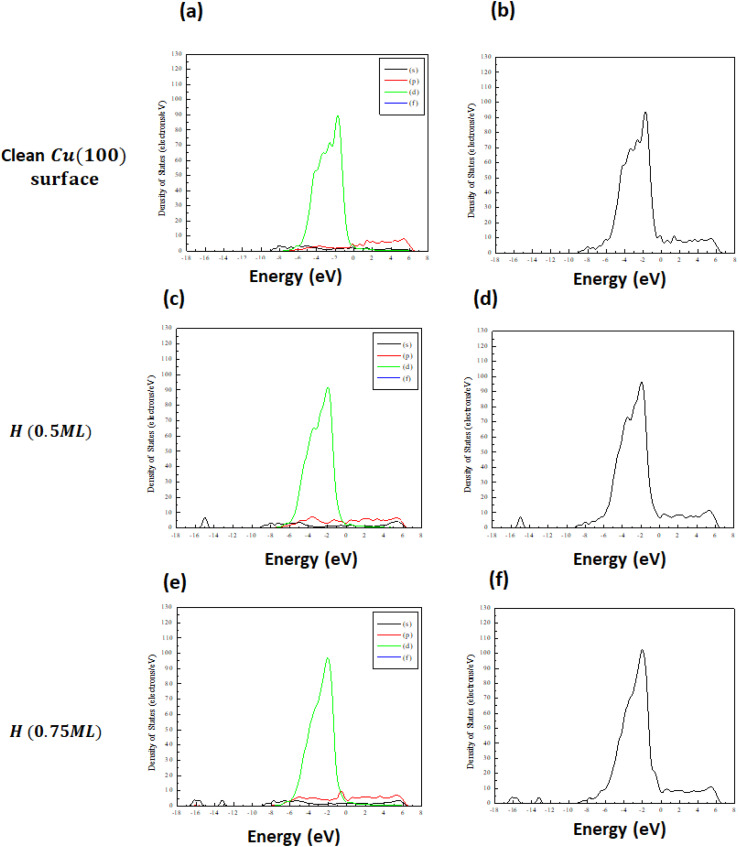
Projected density of states (, left panels) and total density of states (right panels) for (a and b) the clean (100) surface, (c and d) the (100) surface with 0.5 ML coverage, and (e and f) the (100) surface with 0.75 ML coverage.

The projected electronic density of states for bromine adsorption on the Cu(100) surface is illustrated in Fig. 7. This includes: (a and b) a clean Cu(100) surface, (c and d) bromine adsorption in hollow sites at a coverage of 0.5 ML), and (e and f) bromine adsorption in hollow sites at a coverage of 0.75 ML. For the clean Cu(100) surface, as depicted in Fig. 7(a and b), the density of states is characterized predominantly by a sharp, well-defined Cu 3d band occurring mainly below the Fermi level. This feature is typical of metallic copper and is characterized by a narrow bandwidth, minimal external perturbations and a low density of states at the Fermi level. The lack of states near the Fermi level corresponds with the relatively inert properties of clean Cu surfaces.

Fig. 7(c and d) depict the projected electronic density of states (DOS) for a Cu(100) surface with bromine atoms adsorbed at hollow sites at a coverage of 0.5 ML. The plots show significant changes in the density of states relative to the clean Cu(100) surface, suggesting electronic interactions between the Cu substrate and the adsorbed Br atoms. A notable characteristic is the broadening of the Cu 3d band, which becomes less defined in comparison to the more distinct peaks seen on the clean surface. The expansion of the d-band center accompanied by a slight decrease in energy, coupled with hybridization between Cu 3d states and Br 4p orbitals, indicates the formation of Cu–Br bonds.

The electronic states of Br, particularly the 4p orbitals, contribute new features in the DOS, which are most evident in the form of small peaks close to the Fermi level. A noticeable redistribution of electronic states near the Fermi level is observed. Evidence of partial charge transfer is observed from the Cu surface atoms to the Br adsorbates, resulting in a change to the local electronic environment. The charge transfer observed here is a characteristic feature of chemisorption and indicates a moderately strong interaction between Br and the Cu(100) surface at this intermediate coverage. The proximity of states to the Fermi level suggests a rise in electronic activity, potentially impacting the surface's catalytic or electrochemical properties. At a relatively low coverage of 0.5 ML, the adsorption of Br can significantly alter the electronic structure of the Cu(100) surface, thereby creating a foundation for more substantial effects at higher coverages.

Fig. 7(e and f) present the projected electronic density of states (DOS) for the Cu(100) surface with bromine adsorbed at hollow sites at a higher coverage of (0.75 ML). The DOS reveals more substantial modifications, highlighting the increasingly strong interaction between Br atoms and the Cu substrate as coverage increases. A significant observation is the further broadening and downward shift of the Cu 3d band, which reflects enhanced hybridization between Cu 3d and Br 4p orbitals. The intensity and spread of the DOS features indicate stronger and more extensive orbital overlap, suggesting that Br atoms are increasingly perturbing the local electronic structure of the surface. This effect is likely due to the cumulative influence of a denser Br overlayer and closer Br–Br interactions, which intensify the charge redistribution dynamics.

Moreover, the presence of Br at H (0.75 ML) leads to more pronounced Br-derived features in the DOS, particularly from the Br(4p) orbitals. These peaks, coupled with the modification of Cu states, reflect a highly interactive system where the electronic contributions from both substrate and adsorbate are strongly entangled. Overall, the projected DOS analysis reveals that Br adsorption substantially modifies the electronic environment of the Cu(100) surface, particularly at higher coverages. These electronic alterations are consistent with stronger Br–Cu bonding and more extensive charge redistribution, emphasizing the significant role of adsorbate coverage in tuning surface properties.

### 3.4. Surface energetics of Br/Cu(100)

**Fig. 8:**
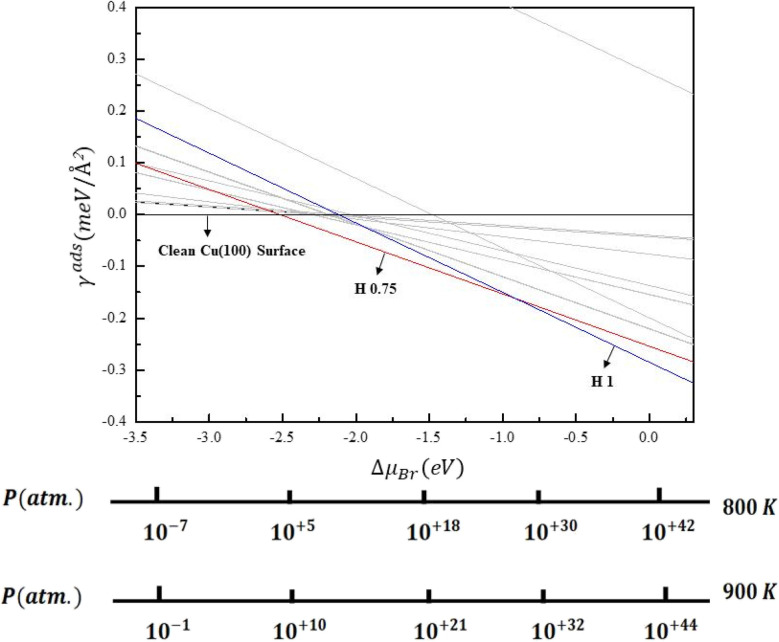
Surface free energies (*γ*_ads_) related to chemical potentials (Δ*μ*_Br_) of various Br/Cu(001) structures. The corresponding pressure is given for temperatures of 800 and 900 K.

The surface free energy is depicted as a function of the bromine chemical potential as shown in Fig. 8. Pressure-temperature bar lines for *T* = 800 and 900 K are overlaid on the diagram to correlate chemical potential with actual experimental conditions (pressure and temperature). This mapping is essential as the bromine chemical potential contingent upon these environmental conditions, influencing the preferred adsorption mode. The phase diagram depicts the thermodynamic stability of each adsorption mode (hollow, bridge, and top) representing distinct binding configurations of bromine atoms on the Cu(001) surface, which reflects their stability under different conditions. The surface free energies are calculated by integrating temperature pressure utilizing *E*_b_ as shown in Table 2. A lower surface free energy signifies a more thermodynamically stable configuration. The transitions or crossovers in the plotted lines indicate alterations in stability among the hollow, bridge, and top modes as the chemical potential or coverage varies.

The phase diagram demonstrates that the clean Cu(100) surface is the most stable structure in a low Br environment [ Δ*μ*_Br_ < −2.30 eV]. As the bromine concentration increased, −2.30 eV to −1.00 eV, the H (0.75 ML) structure exhibited greater stability. This chemical potential corresponds to a temperature and pressure of *T* = 800 K for *P* ranges from 10^+14^ to 10^+30^ atm. or *T* = 900 K for *P* ranges from 10^+18^ to 10^+32^ atm.

This suggests that at intermediate bromine concentrations, the surface becomes partially saturated, with bromine atoms preferentially occupying the energetically favorable hollow sites. Also, as bromine concentration increased further, (>−1.00 eV), the H (1 ML) structure exhibited greater stability. This chemical potential corresponds to a temperature and pressure of *T* = 800 K for *P* > 10^+30^ atm. or *T* = 900 K for *P* > 10^+32^ atm.

Results of the bromine adsorption on the Cu(100) can be applied to environmental chemistry processes that involve surface-mediated halogenation reactions, especially in designing and fine-tuning catalytic and corrosion-resistant materials, as well as in understanding interactions between pollutants and surfaces, which is pertinent to both atmospheric and industrial settings. Understanding bromine's behavior on copper surfaces under varying temperature and pressure conditions facilitates the prediction and control of surface reactions that are crucial to environmental remediation technologies and sustainable industrial processes.

## 4. Conclusions

A comprehensive investigation of bromine adsorption on the Cu(100) surface using density functional theory (DFT) with van der Waals corrections and *ab initio* atomistic thermodynamics was performed. This study explored the structural, energetic, and electronic properties of Br/Cu(100) systems at various adsorption sites (top, bridge, and hollow) and coverages ranging from (0.11 to 1) ML. The binding energy analysis revealed that the hollow site is the most favorable across most coverage levels, with the configuration at 0.75 ML offering the most favorable coverage. van der Waals interactions were shown to significantly enhance the adsorption strength, especially at low coverages, emphasizing the importance of including dispersion corrections in surface studies.

Electronic density of states (DOS) analysis demonstrated significant modifications in the Cu(3d) bands and the appearance of Br(4p) states near the Fermi level, indicative of strong Br–Cu hybridization. Surface free energy calculations and the resulting thermodynamic phase diagram provided clear insight into the relative stability of the adsorption configurations as a function of bromine chemical potential. This work delivers valuable atomic-scale understanding of bromine–copper interactions.

## Conflicts of interest

There are no conflicts to declare.

## Data Availability

The authors declare that all data supporting the findings of this study are available from the corresponding author upon reasonable request.
